# Effects of public reporting of prescription indicators on patient choices: evidence from propensity scores matching

**DOI:** 10.3389/fphar.2023.1110653

**Published:** 2023-10-09

**Authors:** Manli Chen, Xinping Zhang, Chaojie Liu, Haihong Chen, Dan Wang, Chenxi Liu

**Affiliations:** ^1^ School of Management, Hubei University of Chinese Medicine, Wuhan, Hubei, China; ^2^ Research Center for the Development of Traditional Chinese Medicine, Key Research Institute of Humanities and Social Sciences of Hubei Province, Wuhan, China; ^3^ School of Medicine and Health Management, Tongji Medical School, Huazhong University of Science and Technology, Wuhan, Hubei, China; ^4^ School of Psychology and Public Health, La Trobe University, Melbourne, VIC, Australia; ^5^ School of Health Policy and Management, Nanjing Medical University, Nanjing, China

**Keywords:** public reporting, health providers’ performance, physicians, the propensity score method, primary care, antibiotics prescription

## Abstract

**Background:** Public reporting on health providers’ performance (PRHPP) is increasingly used for empowering patients. This study aimed to test the effect of PRHPP using the theory of the consumer choice model.

**Methods:** The study was conducted in 10 primary care institutions in Hubei province, China. Information related to the percentage of prescriptions requiring antibiotics, the percentage of prescriptions requiring injections, and average costs per prescription for each prescriber was calculated, ranked and displayed in a public place on a monthly basis. A questionnaire survey was undertaken on 302 patients 10 months after the initiation of the PRHPP, tapping into patient awareness, understanding, perceived value and use of the information in line with the theory of the consumer choice model. The fitness of data with the model was tested using structural equation modelling. The patients who were aware of the PRHPP were compared with those who were unaware of the PRHPP. The propensity score method (considering differences between the two groups of patients in age, gender, education, health and income) was used for estimating the effects of the PRHPP.

**Results:** About 22% of respondents were aware of the PRHPP. Overall, the patients showed limited understanding, perceived value and use of the disclosed information. The data fit well into the consumer choice model. Awareness of the PRHPP was found to be associated with increased understanding of the antibiotic (*p* = 0.028) and injection prescribing indictors (*p* = 0.030). However, no significant differences in perceived value and use of the information (*p* > 0.097) were found between those who were aware and those who were unaware of the PRHPP.

**Conclusion:** Although PRHPP may improve patient understanding of the prescribing performance indicators, its impacts on patient choices are limited due to low levels of perceived value and use of information from patients. Additional support is needed to enable patients to make informed choices using the PRHPP.

## 1 Introduction

Over the past few decades, public reporting of health providers’ performance (PRHPP) has been increasingly used for the purpose of improving the quality of patient care (Marshall et al., 2000). It started in the United States in the 1980s (Hannan et al., 1994) and has since been adopted by many other developed countries (Hibbard et al., 2003; Marshall et al., 2003). PRHPP is considered as an instrument that can improve the quality of care through enhancing transparency and accountability (Lansky, 2002; Hibbard et al., 2003; Fung et al., 2008). It is expected that patients use the information to choose healthcare providers, which would force healthcare providers to improve their underperforming areas in order to maintain their market share (Berwick et al., 2003; Asch et al., 2006; Fung et al., 2008).

Empirical studies showed that patients are interested in information related to quality of care (Longo and Everet, 2003; Boscarino and Adams, 2004; Cheng and Song, 2004; Sofaer et al., 2005), and nearly 50% consider quality as the most important aspect when choosing a healthcare provider (Robinson and Brodie, 1997; Schneider and Epstein, 1998). However, evidence about the association between PRHPP and patient choices has been inconclusive (Faber et al., 2009; Totten et al., 2012).

It is not clear how patients value and use PRHPP (Marshall et al., 2000; Werner and Asch, 2005; Faber et al., 2009). Hibbard and others proposed a theory of the consumer choice model (CCM), which assumes that the impacts of PRHPP take place through four stages in a sequential order (Hibbard et al., 2002). At the first stage, patients become aware of the PRHPP. Gradually, they develop an appropriate understanding of the information involved in the PRHPP (stage two). But before they use PRHPP as an instrument for choosing healthcare providers (stage four), they have to develop an appreciation of the value of PRHPP (stage three). The perceived value of PRHPP is critical and subject to the influences of both personal and environmental factors, such as the characteristics of patients, the interactive dynamic between patients and healthcare providers, and the healthcare system arrangements.

Faber and others, in a systematic review, identified the lack of understanding of the staged effects of PRHPP on patients as a gap in the literature that deserves attention (Faber et al., 2009). Several studies attempted to understand how patients might use PRHPP in simulated environments (Hibbard et al., 1996; Hibbard et al., 2000; Hibbard et al., 2001a; Uhrig et al., 2006; Peters et al., 2007). A few researchers observed how healthy consumers chose a new health plan using reported performance information on various plans (Knutson et al., 1998; Farley et al., 2002a; Farley et al., 2002b; Hibbard et al., 2002). But there is a paucity in the literature documenting how patients choose a provider when they are ill. In addition, all of the existing studies have been conducted in the USA (Faber et al., 2009). Little is known about the effects of PRHPP in other countries, especially in developing countries.

In this study, we applied the propensity score method (PSM) to estimate the effects of PRHPP on patient changes in line with the CCM theory in Hubei province, China. PSM has been increasingly used in health services research (Austin, 2008), which allows us to estimate causal effects based on cross-sectional data (Caliendo and Kopeinig, 2008).

## 2 Materials and methods

This study was undertaken in 10 primary care institutions in Qianjiang city in China’s Hubei province. A cross-sectional questionnaire survey was conducted on patients who visited the participating institutions, 10 months after PRHPP interventions were introduced.

### 2.1 Study setting

Qianjiang is situated in central Hubei of China, with a population of 0.95 million. Its economic development ranks in the middle range of all cities in Hubei. On average, about 500,000 outpatient visits were recorded per year in Qianjiang. At the time of the study, there was no restriction imposed by the social health insurance programs on patient choices of providers, either at the institutional level or at the individual physician level.

More than 50% of prescriptions prescribed in primary care institutions in Hubei contained antibiotics or injections (Liu et al., 2015; Liu et al., 2016), much higher than the levels recommended by the World Health Organisation (≤30% for antibiotics; ≤24% for injections) (Li, 2014). In November 2013, the Qianjiang health authority introduced a PRHPP program in 10 primary care institutions (the participating institutions of this study), with an aim to curb over-prescriptions. The information released to the public was selected based on the WHO indicators in relation to the rational use of medicines: percentage of prescriptions requiring antibiotics; percentage of prescriptions requiring injections; and average expenditure of medicines per prescription (Laing et al., 1993). The three indicators were calculated and ranked at the individual physician and institutional levels.

They were printed out and displayed in the entrance hall of each participating primary care institution, along with an explanation about the purpose of the PRHPP. Except the information of the three indicators (percentage of prescriptions requiring antibiotics, percentage of prescriptions requiring injection, and average expenditure of patients), the rankings of the three indictors for each physician were also provided for patients. In addition, adverse effects of irrational use of antibiotics (such as prolonged hospital stay and increased medical expenditure) was also provided for helping consumers understand the information at the footnote of the displayed poster. An example of the displayed poster is provided in the supplementary file. The displayed information was updated on a monthly basis: 49%–71% prescriptions contained antibiotics; 50%–64% prescriptions contained injections; average expenditure per prescription ranged from ¥38 to ¥55 (roughly $5.5–8.0). Details about the design of the PRHPP interventions were published (Du et al., 2015).

### 2.2 Study instrument

The questionnaire was adapted from Hibbard’s survey instrument (Hibbard et al., 2002). It contained 10 items measuring four domains in line with the CCM theory: awareness, understanding, perceived value and use of information. Patient awareness of PRHPP was captured by one item, asking the respondents whether they had read the PRHHP poster (yes or no). Each of the domains measuring patient understanding, perceived value and use of information contained three items, focusing on antibiotics, injections and costs of prescriptions, respectively. The respondents were asked to rate each item on a five-point Likert scale, with a higher score indicating a more positive response.

The validity of the instrument was tested using exploratory factor analysis (EFA) with varimax rotation, and structural equation modelling (SEM). The EFA produced three factors (excluding the item measuring patient awareness) as expected, with items loading highly and exclusively on their corresponding factors. By testing a model consisting the three latent factors (awareness, understanding, perceived value and use of information) using item parcels as indicators per latent variable, SEM was applied for testing construct validity of the instrument (Keller et al., 1998). The SEM indicated that the CCM had a good fitness with data: RMSEA = 0.051 (<0.08), SRMR = 0.057 (<0.08) and CFI = 0.991 (>0.95) (Hooper et al., 2008).

Data collected for PSM included age, gender, educational attainment, self-rating on health, and income. These variables were selected based on a systematic review (Faber et al., 2009). Self-rating on health was measured using a five-point Likert scale. Annual household average income was estimated with 11 equal intervals ranging from less than 10,000 Yuan (US$1,500) to over 100,000 Yuan (US$15,000). We did not collect diagnostic information because more than half of the respondents completed the questionnaires prior to medical consultations. A translated version of instrument is provided in the supplementary file.

### 2.3 Sample size

We estimated sample size based on the following formula:

$$n=\frac{{\left(Z_{\alpha /2}+Z_{\beta }\right)}^{2}*2*\sigma^{2}}{d^{2}}$$

A previous study showed that PRHPP could lead to 0.76 changes in CCM scores (Hibbard et al., 2002). A sample size of 63 (for each group) would enable us to detect a difference (d) of 0.5 in CCM scores (standard deviation *σ* = 1.0) between those who were aware and those who were unaware of the PRHPP, with a probability of type one error *α* = 0.05 and statistical power *β* = 0.8. If 25% of patients became aware of the PRHPP, a minimal of 252 participants would be needed. We increased the sample size to 300.

### 2.4 Sampling and data collection

Data were collected from the 5th to 8th August 2014, 10 months after the introduction of the PRHPP. Patients who visited the outpatient clinics of the participating primary care institutions were eligible to participate in the survey. About ten or more physicians were made available for patients in the participating primary care institutions at any time of patient visits. For patients younger than 18 years, their parents were surveyed. Adult patients who were unable to read or communicate were excluded from this study.

A systematic sampling strategy was adopted. About 30 questionnaires were collected in each participating primary care institution. It was estimated that about 100 patients visited each outpatient clinic per day. Thus, one in every three patients was invited to participate in the survey. If less than 30 questionnaires were returned in a participating clinic, additional questionnaires were collected the next day.

The questionnaires were administered through face-to-face interviews in the patient waiting areas. Each clinic was attended by two interviewers. Four pairs of postgraduate students were trained to conduct the survey. The returned questionnaires were checked for completeness by XP on the day of the survey.

We planned to distribute 350 questionnaires, and ended up with a final sample size of 302 (86.29%) for data analyses.

### 2.5 Statistical analysis

The items measuring patient understanding, perceived value and use of information were given a score, ranging from 1 to 5 (with a higher score indicating a more positive response). Shapiro-Francia W′ tests were performed to determine the normality of data distributions. Two-sample independent t tests (for data with normal distributions) or Kruskal–Wallis tests (for data with non-normal distributions) were performed to compare the differences in the scores between the respondents who were aware and those who were unaware of the PRHPP.

We also transformed the scores into dichotomous measurements using a cut-off value: >3 indicating a positive response. Chi-square tests were applied to test the differences between those who were aware and those who were unaware of the PRHPP.

#### 2.5.1 SEM

We established SEM to test the following hypothesis based on the CCM (Hibbard et al., 2002):

• Hypothesis 1: patient awareness of the PRHPP improved their understanding of the prescribing performance information.
• Hypothesis 2: better patient understanding about the prescribing performance information improved their perceived value of the information.
• Hypothesis 3: higher perceived value of the prescribing performance information increased patient use of the information.

In the SEM, patient awareness was treated as an observable variable, while patient understanding, perceived value and use of information were treated as latent variables, each being measured by three observable variables. The standardized structural coefficients were calculated to test the links between those variables.

#### 2.5.2 Effect estimation using PSM

PSM allows researchers to identify matched control groups for estimating the effect size of a certain treatment (such as patient awareness of the PRHPP in this study). It reduces the bias resulting from a lack of distribution overlap between two compared groups, a bias that cannot be detected by regression analyses (Li, 2013). PSM involves three steps: 1) calculating propensity scores based on observational variables that have a significant impact on the estimated results; 2) matching participants in the treatment and control groups based on propensity scores; 3) estimating the effect of treatment based on matched samples.

In this study, propensity scores were calculated using a logistic regression model, considering the distributions of age (elderly/none-elderly), gender (male/female), education (primary school, secondary school, high school, college), self-rated health (good, medium, poor), and family income (<50,000 Yuan or ≥50,000 Yuan) for those who were aware and unware of the PRHPP. These covariates were identified based on a systematic review (Faber et al., 2009). No-replacement one-to-one nearest-neighbor matching was applied to form a matched sample based on the propensity scores. The caliper of matching was set as 0.03 (Austin, 2009). The differences between the paired participants served as the basis for estimating the effects of the PRHPP: average treatment effect (ATE) and average treatment effect on the treated group (ATT) (Li, 2013):

$$\text{ATE}=E\left(Y_{1i}|T_{i}=1,0\right)-E\left(Y_{0i}|T_{i}=1,0\right)$$

$$\text{ATT}=E\left(Y_{1i}|T_{i}=1\right)-E\left(Y_{0i}|T_{i}=1\right)$$

In these formula, E(.) indicates the expectation in the population. $Y_{1i}$ and $Y_{0i}$ are potential effects of the PRHPP on individual i when i is aware of the PRHPP ( $Y_{1i}$ ) or is not aware of the PRHPP ( $Y_{0i}$ ). $T_{i}$ represents PRHPP with 1 indicating the participants who were aware of the PRHPP and 0 indicating those who were unaware of the PRHPP. ATE refers to an average effect that would be observed if all participants were aware of the PRHPP compared with that if none was aware of the PRHPP. ATT refers to an average effect difference that would be found if the participants who were aware of the PRHPP became unaware of the PRHPP (Li, 2013).

A *p*-value of <0.05 was considered as significant and all analyses were performed using STATA 12.0.

## 3 Results

### 3.1 Demographic characteristics of participants

About half (48%) of the respondents were women; less than 13% were 65 years of age or older; over three-quarters completed no more than primary or secondary school; and the majority (80%) had an annual household income under ¥50,000 (US $7264).

Only 21.5% of respondent were aware of the PRHPP. Non-significant differences between those who were aware and those who were unaware of the PRHPP existed in the demographic characteristics (Table 1).

**TABLE 1:** Characteristics of participants (N, %).

Characteristics		Total	Unaware of PRHPP	Aware of PRHPP	*p*-value*
Number of participants		302	237 (78.48)	65 (21.52)	-
Women		145 (48.01)	119 (50.21)	26 (40.00%)	0.144
Elderly		38 (12.58)	33 (13.92)	5 (7.69)	0.180
Education	Primary school	96 (31.79)	83 (35.02)	13 (20.00)	0.092
	Secondary school	133 (44.04)	102 (43.04)	31 (47.69)	
	High school	60 (19.87)	42 (17.72)	18 (27.69)	
	College and above	13 (4.30)	10 (4.22)	3 (4.62)	
Self-rated Health	Good	183 (60.60)	142 (59.92)	41 (63.08)	0.733
	Fair	81 (26.82)	66 (27.85)	15 (23.08)	
	Poor	38 (12.58)	29 (12.24)	9 (13.85)	
Family income	<¥50,000	242 (80.13)	192 (81.01)	50 (76.92)	0.464
	≥¥50,000	60 (19.87)	45 (18.99)	15 (23.08)	

*p-value was calculated using chi-square tests.

### 3.2 Findings of SEM

The three hypotheses were supported by the SEM results (Table 2): patient awareness of PRHPP led to better understanding (coefficient = 0.291, *p* < 0.001) of prescribing performance indicators; better understanding increased perceived value (Coefficient = 0.342, *p* < 0.001) of the information; higher perceived value increased use of the information (Coefficient = 0.692, *p* < 0.001).

**TABLE 2:** Results of structural equation modelling on the consumer choice model.

Relationship/index		Coefficients (standardized error) *	*p*-value
Structural relationship	Awareness → understanding	0.291 (0.055)	<0.001
	Understanding → perceived value	0.342 (0.056)	<0.001
	Perceived value → use	0.692 (0.032)	<0.001
Model fit index^#^	RMSEA	0.051	-
	SRMR	0.057	-
	CFI	0.991	-

*values have been standardized.

^#^RMSEA, root mean square error of approximation; SRMR, standardized root mean square residual; CFI, comparative fit index.

### 3.3 Effects of PRHPP

The respondents reported limited understanding, perceived value and use of the prescribing performance information, with a mean score ranging from 2.04 to 2.95 out of a possible 5 (Table 3). Those who were aware of the PRHPP had higher scores and a higher percentage of positive responses compared with those who were unaware of the PRHPP in the following aspects: understanding of prescribing indicators associated with antibiotics (*p* < 0.001) and injections (*p* < 0.001) and use of the injection indicator (*p* < 0.05). The patients who were aware of the PRHPP were also more likely to have a positive response to the use of the antibiotic indicator (*p* = 0.001), despite a lack of significant difference in the mean scores (*p* = 0.08).

**TABLE 3:** Patient responses to the PRHPP.

Patient response		Overall		Unaware		Aware		*p*-value*	
		Mean (SD)	Positive response (%)	Mean (SD)	Positive response (%)	Mean (SD)	Positive response (%)	Mean	Positive response
Understanding of the performance information	Antibiotics	2.12(1.10)	17.88	1.97(1.04)	13.92	2.65(1.14)	32.31	<0.001	0.001
	Injections	2.06(1.05)	14.57	1.91(0.99)	10.55	2.61(1.10)	29.23	<0.001	<0.001
	Costs	2.04(1.01)	12.91	2.00(1.04)	14.35	2.17(0.91)	7.69	0.086	0.156
Perceived value of the performance information	Antibiotics	2.71(1.11)	28.48	2.64(1.09)	27.00	2.93(1.14)	33.85	0.055	0.279
	Injections	2.75(1.11)	29.47	2.69(1.08)	27.85	2.97(1.19)	35.38	0.073	0.238
	costs	2.80(1.09)	29.47	2.78(1.07)	30.80	2.86(1.13)	24.62	0.595	0.332
Use of the performance information	Antibiotics	2.90(1.06)	30.13	2.85(0.98)	25.74	3.11(1.30)	46.15	0.080	0.001
	Injections	2.91(1.06)	32.12	2.85(0.99)	26.58	3.15(1.25)	52.31	0.039	<0.001
	Costs	2.95(1.04)	31.79	2.92(0.99)	29.11	3.05(1.22)	41.54	0.388	0.057

*p-value was calculated using chi-square test in positive response comparison and two-sample *t*-test or Kruskal–Wallis test in mean comparison, based on normality of dependent variables.

The effects estimated using PSM showed that the PRHPP led to a 0.5 increase in ATE (*p* < 0.05) and a 0.6 increase in ATT (*p* < 0.001) with regard to patient understanding of the prescribing performance information associated with antibiotics and injections (Table 4). However, no significant effects were found on the other aspects of the CCM (*p* > 0.1).

**TABLE 4:** Effects of the PRHPP estimated using PSM.

Effects of PRHPP		ATE*		ATT^#^	
		*Coefficient*	*p*-value	*Coefficient*	*p*-value
Understanding of performance information	Antibiotics	0.445	0.028	0.623	<0.001
	Injections	0.538	0.030	0.611	<0.001
	Costs	0.180	0.133	0.203	0.166
Perceived value of performance information	Antibiotics	−0.012	0.917	0.108	0.539
	Injections	−0.063	0.609	0.084	0.636
	costs	−0.199	0.097	−0.103	0.544
Use of performance information	Antibiotics	−0.009	0.954	0.119	0.579
	Injections	0.022	0.880	0.154	0.461
	Costs	−0.129	0.375	0.038	0.856

*ATE, average treatment effect.

^#^ATT, average treatment effect on treatment group.

## 4 Discussion

This study revealed that PRHPP can improve patient understanding about the prescribing performance information, but it failed to translate into a useful tool to help patients make choices. Previous studies also showed no or weak evidence to support the link between PRHPP and patient choices in real world contexts (Faber et al., 2009; Totten et al., 2012), despite strong evidence supporting such a link in an experimental environment.

PRHPP serves as one way to inform consumer healthcare choices by comparing physicians’ performance of various dimensions of quality and cost. PRHPP practice in this study covered the core prescribing indicators of percentage of prescriptions requiring antibiotics, percentage of prescriptions requiring injection and cost indictor of average expenditure of patients. WHO in collaboration with the International Network of Rational Medicine Use (INRDU) recommended the set of core prescribing indictors, which have been widely applied for evaluating antibiotic prescribing quality among primary healthcare facilities in developing countries (Aravamuthan et al., 2017; Amaha et al., 2019; Kilipamwambu et al., 2021).

Based on the results from a recent review, public reporting of physicians’ and hospitals’ performance can help stimulate quality improvement, inform consumer choices and ultimately improve clinical results moderately. For consumers, with transparent and easily available performance information, PRHPP could help facilitate consumers to select a physician or medical institutions that appeared to have better results (Prang et al., 2021). However, whether PRHPP works or how the amount of practical effects depended on a list of various factors, such as the appropriateness of disseminated channel of PRHPP, relevance and meaningfulness of the chosen indictors, and consumer characteristics, which are discussed below for the limited effects of PRHRR in this study (Hibbard and Sofaer, 2010).

The CCM theory postulates a staged process for behavioral changes. It is essential to make sure that the reported information is valuable from the perspective of consumers before they are willing to use the information for decision making. Although in this study, participants enjoyed the freedom to choose providers, they might not necessarily appreciate the value of the PRHPP information for several reasons.

Firstly, the overuse of antibiotic and injection prescriptions is common and consistently high in primary care institutions and prescription costs have already been lowered due to the recent health system reform in China. Empirical studies show that PRHPP is more useful for patients when obvious differences in provider behaviors are observable (Harris, 2002). Otherwise, little value would be perceived by patients in relation to changing providers. Indeed, the percentage of prescriptions requiring antibiotics or injections are overwhelmingly high in China (Liu et al., 2015). Despite strong government interventions to tackle this problem, there has been no sign of decline in antibiotic and injection prescriptions (Li, 2014; Liu et al., 2015; Liu et al., 2016). In the participating institutions of this study, no prescriber was able to meet the WHO recommendations in relation to of the rational use of medicines (Laing et al., 1993).

Secondly, consumer demand for antibiotics and injections are high. In China, many patients believe that antibiotics and injections are a shortcut to quick recovery from many illnesses, including common colds (Wei et al., 2006). Such a misunderstanding may seriously jeopardize the value of PRHPP (Baker et al., 2014; Spaling et al., 2015; Dodds et al., 2016). The misconception could negatively affect the perception of the raking indictors of PRHPP practice. It was likely that PRHPP may lead more patients to seek services from those doctors prescribed more antibiotics and injections. Numerous studies have demonstrated that a lack of appropriate understanding is a major barrier preventing patients from using PRHPP information (Hibbard and Jewett, 1996; Jewett and Hibbard, 1996; Boscarino and Adams, 2004; Richard et al., 2005; Sofaer et al., 2005; Robinowitz and Dudley, 2006). Although we found significant improvement in patient understanding of the PRHPP information, such improvement is limited. Some researchers recommend simplified presentations such as a star rating to improve understanding and endorsement from patients (Peters et al., 2007; Damman et al., 2012).

Thirdly, the disclosed information may not be considered relevant to the priorities of the patients. Patients are more likely to appreciate the information that fits better with their needs. For example, cancer patients would need quite different information compared to diabetic patients (Edgman-Levitan and Cleary, 1996). Unfortunately, we were not able to collect diagnostic information because more than half of the questionnaires were collected prior to medical consultations.

Finally, the level of patient awareness of the PRHPP is low. Only one-fifth of the respondents reported being aware of the PRHPP. This rate is much lower compared with those (49%–78%) found in studies conducted in the USA (Knutson et al., 1998; Farley et al., 2002a; Hibbard et al., 2002). However, it is important to acknowledge that these studies offered participants a choice of a healthcare plan which is relevant to almost everyone (Knutson et al., 1998; Farley et al., 2002a; Farley et al., 2002b; Hibbard et al., 2002). For information related to patient care interventions, however, it is challenging to attempt to attract attention from all patients. Some researchers argued that patients with different illness conditions may have very different preferences in the choice of medical interventions. The rationale behind their choices could not be understood through observations of choices made by healthy people (Faber et al., 2009). Schneider and Epstein reported that only 12% of hospital patients who underwent coronary artery bypass grafting (CABG) surgery paid attention to reported CABG mortality rates (Schneider and Epstein, 1998). It is not clear why so many CABG patients ignored the reported mortality information.

A lack of understanding and support from patients for the rational use of medicines is a serious issue of concern. The overuse of antibiotics and injections is very common in China and many other developing countries. It has contributed to the rapid spread of antibiotic resistance (Robinowitz and Dudley, 2006), transmission of the human immunodeficiency virus and hepatitis B virus (Damman et al., 2012), and escalation of medical costs.

PRHPP is intended to be an instrument for empowering patients. However, careful design of the PRHPP is essential to obtain endorsement from patients. A number of guidelines are available for developing a readable and understandable PRHPP (Drozda et al., 2008; Hussey et al., 2014).

New approaches should be developed to solve the effectiveness of PRHPP practice, such as engagement of professional societies, increasing disseminating channel of public information, determining the best format for presentation of information to consumers, and eliminating the misconceptions of the information by consumers (Dehmer et al., 2014). The engagement of professional societies has been mentioned as one effective way for developing meaningful performance measures and promoting use of public reporting. Increasing the disseminating channel is also identified as one effective strategy, for example, information can be reported through a range of media, such as individual reminders, educational materials, public forums, clinical audits and feedback (Grimshaw et al., 2004). Research evidence shows that explanatory messages are not effective in correcting misconceptions and in increasing the perceived value of PRHPP (Hibbard et al., 2000). A simple message about risks may be more effective. A study conducted in India suggests that information disseminated from physicians is more effective than public reporting, resulting in lower injection use (Bhunia et al., 2010), possibly because the information targeted those with the highest risk. General education of the public plays an important role in the effectiveness of PRHPP. Low levels of education and socioeconomic status are usually associated with low awareness and poor understanding of PRHPP (Hibbard et al., 2001b). The average level of education and income of the participants of this study was low, which might have contributed to the limited effects of the PRHPP.

The effects of PRHPP should be explained with caution. The effects of the PRHPP, both positive and negative, are context dependent. In this study, three prescription indicators were reported in line with the WHO recommendations (Laing et al., 1993). Similar to the findings of other studies (Robinson and Brodie, 1997; Schneider and Epstein, 1998), these three indicators did not attract equal attention from patients: patients are more concerned about the quality rather than the cost of prescriptions. However, given the prevalent misconception about antibiotic and injection interventions from consumers, there is a risk that PRHPP may encourage more patients to seek services from those doctors who are more likely to prescribe antibiotics and injections. In addition, the doctors who rank low in the league table may choose to increase antibiotic and injection prescriptions in order to increase their market share (Wang et al., 2014). Some researchers worry that the reported poor performance of health workers may exacerbate the existing strained relationship between physicians and patients in China (Tang et al., 2008), stimulating distrust and more defensive practices (Totten et al., 2012). Further studies are needed to tap into these questions.

This study has made a significant contribution to the literature by applying CCM theory to explore the effects of PRHPP in a real-world context. The SEM analysis showed that the CCM theory fits well with the data. The application of PSM reduced potential bias in estimating the effect size of the PRHPP (Li, 2013).

### 4.1 Limitation

There were several limitations in this study. First, the data were collected 10 months after the introduction of the PRHPP, which may not be long enough to detect the effects of PRHPP, especially for those at a later stage of the CCM. However, the immediate effect of PRHPP on patient understanding of prescription information is still promising. On the other hand, the effects of PRHPP on informing patient choices is limited and further measures targeting on improving effectiveness of PRHPP should be considered in future studies. Third, the participants of this study had relatively low socioeconomic status, which may result in under-estimation of the effects of PRHPP. Finally, the survey was undertaken in primary care institutions in Hubei province. Generalization of the results to other facilities and regions needs to be cautious.

## 5 Conclusion

Patient awareness of the PRHPP is low. The effects of the PRHPP are limited, with some improvement of patient understanding about the antibiotic and injection prescription indicators. The PRHPP failed to show significant impacts on patient perceived value and use of information. The healthcare system contexts and low socio-economic status of the participants may be associated with the limited effects of the PRHPP. Appropriate patient education and provider training are prerequisites for the introduction of PRHPP programs.

## Data Availability

The raw data supporting the conclusion of this article will be made available by the authors, without undue reservation.

## Abbreviations

PRHPP, Public reporting of health providers’ performance; ATE, Average treatment effect; ATT, Average treatment effect on the treated group; CCM, Consumer choice model; PSM, Propensity-score method; SD, Standard deviation; SEM, Structural equation modelling; CABG, Coronary artery bypass grafting.
