# Supplementary material for: Effects of public reporting of prescription indicators on patient choices: evidence from propensity scores matching
Source: Front Pharmacol. 2023 Oct 9;14:1110653. doi: 10.3389/fphar.2023.1110653 (PMC10591321; doi:10.3389/fphar.2023.1110653)
Supplement: Supplementary file 1 [file Table1.DOCX]

**Supplementary File**

**Supplementary Table 1 and Table 2 are examples of displayed poster of public reporting of healthcare providers’ performance**

Supplementary Table 1: Health providers’ performance on rational use of medicine in H primary care institution (2014.3)

| Departments | Name | Percentage of prescriptions requiring antibiotics (%) | Rank | Percentage of prescriptions requiring injection (%) | Rank | Average expenditure of patients(￥ yuan) | rank |
| --- | --- | --- | --- | --- | --- | --- | --- |
| Obstetrics and Gynecology | A1 |  |  |  |  |  |  |
|  | A2 |  |  |  |  |  |  |
|  | A3 |  |  |  |  |  |  |
| Ophthalmology and Otorhinolaryngology | B1 |  |  |  |  |  |  |
|  | B2 |  |  |  |  |  |  |
| Internal Medicine department | C1 |  |  |  |  |  |  |
|  | C2 |  |  |  |  |  |  |
|  | C3 |  |  |  |  |  |  |
| surgical department | D1 |  |  |  |  |  |  |
|  | D2 |  |  |  |  |  |  |
|  | D3 |  |  |  |  |  |  |

Note:

1. Prescribing indicators were calculated using data extracted from the electronic health information system on a monthly basis. All the results were calculated by school of medicine and health management, Tongji Medical College of Huazhong University of Science and Technology.

2. Irrational drug prescribing contributes to bacterial resistance and adverse reaction. Adverse reaction and hospital-acquired infection of antibiotic-resistant bacteria has the capacity to increase medical expenditure, length of hospital stay, and ultimately patient mortality.

Supplementary Table 2: Health providers’ performance on rational use of medicine in 10 primary care institutions (2014.3)

| institutions | Percentage of prescriptions requiring antibiotics (%) | Rank | Percentage of prescriptions requiring injection (%) | Rank | Average expenditure of patients(￥ yuan) | rank |
| --- | --- | --- | --- | --- | --- | --- |
| A primary care institution |  |  |  |  |  |  |
| B primary care institution |  |  |  |  |  |  |
| C primary care institution |  |  |  |  |  |  |
| D primary care institution |  |  |  |  |  |  |
| E primary care institution |  |  |  |  |  |  |
| F primary care institution |  |  |  |  |  |  |
| G primary care institution |  |  |  |  |  |  |
| H primary care institution |  |  |  |  |  |  |
| I primary care institution |  |  |  |  |  |  |
| J primary care institution |  |  |  |  |  |  |

Note:

1. Prescribing indicators were calculated using data extracted from the electronic health information system on a monthly basis. All the results were calculated by school of medicine and health management, Tongji Medical College of Huazhong University of Science and Technology.

2. Irrational drug prescribing contributes to bacterial resistance and adverse reaction. Adverse reaction and hospital-acquired infection of antibiotic-resistant bacteria has the capacity to increase medical expenditure, length of hospital stay, and ultimately patient mortality.

**The following is the translated version of survey instrument:**

**No.______** **Name of Institution:__________**

Patient Instrument regarding to public reporting of health providers’ performance

Ms./Mr.

Greeting.

To improve the quality of care, health providers’ performances on rational use of medicine have been publicly reported. Released rational use of medicine indicators are percentage of prescriptions requiring antibiotics; percentage of prescriptions requiring injections; and average expenditure of medicines per prescription.

The aim of current survey is to investigate patient awareness, understanding, perceived value and use of such reported information. Your personal information will be kept strictly confidential. Thanks very much for your participation.

Tongji Medical School

Huazhong University of Science and Technology, Wuhan

**Note: Please read carefully, and mark the most suitable option for you as “√”.**

**Part 1: Patient awareness, understanding, perceived value and use**

1. Whether you had read the poster regarding health providers’ performance on rational use of medicine displayed in the entrance hall?

🞏Yes 🞏No

1. whether you understand reported indicator named “percentage of prescriptions requiring antibiotics”?

🞏 not understand 🞏understand a bit 🞏partly understand 🞏 understand 🞏 fully understand

1. whether you understand reported indicator named “percentage of prescriptions requiring injections”?

🞏 not understand 🞏understand a bit 🞏partly understand 🞏 understand 🞏 fully understand

1. whether you understand reported indicator named “average expenditure of medicines per prescription”?

🞏 not understand 🞏understand a bit 🞏partly understand 🞏 understand 🞏 fully understand

1. Whether you think physicians’ performance regarding “percentage of prescriptions requiring antibiotics” is valuable for you when choosing health providers?

🞏 not valuable 🞏 valuable a bit 🞏partly valuable 🞏 valuable 🞏 fully valuable

1. Whether you think physicians’ performance regarding “percentage of prescriptions requiring injections” is valuable for you when choosing health providers?

🞏 not valuable 🞏 valuable a bit 🞏partly valuable 🞏 valuable 🞏 fully valuable

1. Whether you think physicians’ performance regarding “average expenditure of medicines per prescription” is valuable for you when choosing health providers?

🞏 not valuable 🞏 valuable a bit 🞏partly valuable 🞏 valuable 🞏 fully valuable

1. Whether you used physicians’ performance regarding “percentage of prescriptions requiring antibiotics” when choosing health providers?

🞏 not used 🞏 used a bit 🞏 partly used 🞏 used 🞏 fully used

1. Whether you used physicians’ performance regarding “percentage of prescriptions requiring injections” when choosing health providers?

🞏 not used 🞏 used a bit 🞏 partly used 🞏 used 🞏 fully used

1. Whether you used physicians’ performance regarding “average expenditure of medicines per prescription” when choosing health providers?

🞏 not used 🞏 used a bit 🞏 partly used 🞏 used 🞏 fully used

**Part 2: Personal information**

**Gender:** 🞏 male 🞏 female **Age:** ____ (Year) **Literacy:** 🞏 primary school 🞏 secondary school 🞏 high school 🞏 college

**Health:** 🞏 very poor 🞏 poor 🞏 medium 🞏 good 🞏 very good

**Average Annual Family Income:** 🞏 <=￥10,000 🞏￥10,000-20,000 🞏￥20,000-30,000 🞏￥30,000-40,000 🞏￥40,000-50,000 🞏￥50,000-60,000 🞏￥60,000-70,000 🞏￥70,000-80,000 🞏￥80,000-90,000 🞏￥90,000-100,000 🞏 >￥100,000
